# Efficacy of Semaglutide in Pediatric Patients With Bardet-Biedl Syndrome and Alström Syndrome

**DOI:** 10.1210/jcemcr/luaf266

**Published:** 2025-11-26

**Authors:** Hajar Dauleh, Idris Mohammed, Khalid Hussain

**Affiliations:** Endocrinology Department, Sidra Medicine, Education City North Campus, PO Box 26999, Al Luqta Street, Doha 00000, Qatar; Endocrinology Department, Sidra Medicine, Education City North Campus, PO Box 26999, Al Luqta Street, Doha 00000, Qatar; Endocrinology Department, Sidra Medicine, Education City North Campus, PO Box 26999, Al Luqta Street, Doha 00000, Qatar

**Keywords:** Alström syndrome, Bardet-Biedl syndrome, semaglutide, obesity, GLP-1 agonist

## Abstract

Bardet-Biedl syndrome (BBS) and Alström syndrome (AS) are rare autosomal recessive ciliopathies characterized by severe multisystemic involvement, including metabolic, sensory, and developmental impairments. Both conditions result from primary cilia dysfunction, disrupting pathways such as leptin and insulin signaling, which leads to obesity and insulin resistance. While setmelanotide, a melanocortin-4 receptor agonist, may be an effective therapy for obesity in these conditions (especially BBS), its cost limits accessibility for many patients. We describe two pediatric cases (one with BBS and another with AS) demonstrating significant metabolic improvements with semaglutide, a glucagon-like peptide-1 receptor agonist. These observations suggest that some cases of BBS and AS may be treated with semaglutide.

## Introduction

Bardet-Biedl syndrome (BBS) and Alström syndrome (AS) are rare ciliopathies caused by homozygous pathogenic variants in genes encoding proteins essential for primary cilia function [1]. These disorders share overlapping phenotypes, including early-onset obesity, insulin resistance, and sensory impairments such as retinal degeneration and hearing loss [2, 3].

BBS is genetically heterogeneous, involving pathogenic variants in at least 22 known genes encoding components of the BBSome complex, which regulates ciliary trafficking [4]. BBS presents with severe early-onset obesity, hyperphagia, insulin resistance, and a predisposition to type 2 diabetes mellitus (T2DM). Retinal dystrophy manifests as night blindness in childhood, progressing to complete vision loss, and hearing loss occurs less frequently. Renal anomalies, including cystic dysplasia and chronic kidney disease [5], are common. Neurological issues, including global developmental delay and autism spectrum disorder, are frequent, along with skeletal abnormalities such as polydactyly and scoliosis.

AS is caused by pathogenic variants in the *ALMS1* gene, which encodes a protein essential for the structure and function of primary cilia. Its clinical presentation includes progressive multisystemic involvement, with early-onset obesity and insulin resistance being hallmarks, often progressing to T2DM during adolescence. Retinal dystrophy leads to early visual loss, typically in infancy, progressing to blindness, while sensorineural hearing loss is another common feature. Cardiac complications, particularly dilated cardiomyopathy, frequently develop early, contributing to considerable morbidity and mortality. Hepatic and renal fibrosis is characteristic, often culminating in organ dysfunction. Growth and developmental delay, short stature, and scoliosis are also frequently observed [3].

Setmelanotide, a melanocortin-4-receptor (MC4R) agonist, is the first US Food and Drug Administration–approved therapy for obesity in ciliopathies such as BBS and AS [6]. Activating the melanocortin pathway downstream of leptin receptor signaling restores appetite regulation and reduces hyperphagia. Clinical trials have demonstrated significant weight loss and improved metabolic outcomes, mostly in BBS patients [7, 8]. However, setmelanotide's prohibitive cost limits its accessibility and widespread use.

In contrast, semaglutide offers a widely available, cost-effective alternative. By directly activating glucagon-like peptide-1 (GLP-1) receptors, semaglutide bypasses ciliary dysfunction and may address the underlying metabolic derangements in BBS and AS.

## Case Presentation

### Case 1: Bardet-Biedl Syndrome

A 7-year-old boy was diagnosed with BBS based on characteristic clinical features, including global developmental delay, hyperphagia, progressive vision loss, and renal abnormalities. On physical examination, he was noted to have microcephaly, with a head circumference at the third percentile, weight above the 99th percentile, and height above the 85th percentile. Notably, acanthosis nigricans was present on the posterior neck and axillae.

### Case 2: Alström Syndrome

A 10-year-old boy was diagnosed with AS based on clinical features, including early-onset obesity, hyperphagia, moderate to severe low vision, and hyperopia with astigmatism. He had bilateral sensorineural hearing loss and used hearing aids. He attended a specialized school due to developmental and sensory impairments. On physical examination, he had truncal obesity and acanthosis nigricans. Cardiac evaluation included electrocardiograms and echocardiogram findings within normal limits.

## Diagnostic Assessment

### Case 1: Bardet-Biedl Syndrome

Whole-exome sequencing identified a nonsense homozygous pathogenic variant c.555G>A, p.Trp185Ter in *BBS21*. This genetic finding confirmed the diagnosis of BBS. Baseline investigations showed elevated LDL, TAG, and TC. ALT was slightly increased, and HbA1c was within the normal range (Table 1).

**Table 1.** Body measurements and laboratory results before treatment and after 3 and 6 months of semaglutide therapy in a patient with Bardet-Biedl syndrome and obesity

	Baseline	3 mo of treatment	6 mo of treatment	9 mo of treatment	Normal reference range
Weight	36.15 kg (SD +2.32)	31.10 kg (SD +1.4)	30.9 kg (SD +1.19)	30.90 kg (SD +1.19)	
BMI	22.1 (SD +2.32)	18.81 (SD +1.47)	18.75 (SD +1.44)	18.09 (SD +1.14)	5th-85th percentile (∼13.5-17.5)
HbA_1c_, %	5.4% (36 mmol/mol)	5.0% (31 mmol/mol)	5.0% (31 mmol/mol)		Normal: <5.7% (<39 mmol/mol) Prediabetes: 5.7-6.4% (39-47 mmol/mol) Diabetes: ≥6.5% (≥48 mmol/mol)
ALT	31 IU/L	20 IU/L	25 IU/L		<25 IU/L
AST	31 IU/L	29 IU/L	27 IU/L		<40 IU/L
TC	5.0 mmol/L (193 mg/dL)	4.4 mmol/L (170 mg/dL)	4.3 mmol/L (166.3 mg/dL)		Acceptable: <4.4 mmol/L (<170 mg/dL) Borderline: 4.4-5.1 mmol/L (170-199 mg/dL) High: ≥5.2 mmol/L (≥200 mg/dL)
TAG	2.5 mmol/L (96.7 mg/dL)	0.6 mmol/L (23.2 mg/dL)	1.2 mmol/L (46.4 mg/dL)		Acceptable: <1.02 mmol/L (<90 mg/dL) Borderline: 1.02-1.46 mmol/L (90-129 mg/dL) High: ≥1.47 mmol/L (≥130 mg/dL)
LDL-C	3.6 mmol/L (139 mg/dL)	2.8 mmol/L (108 mg/dL)	2.7 mmol/L (104.4 mg/dL)		Acceptable: <2.85 mmol/L (<110 mg/dL) Borderline: 2.85-3.34 mmol/L (110-129 mg/dL) High: ≥3.36 mmol/L (≥130 mg/dL)
HDL-C	1.4 mmol/L (54.1 mg/dL)	1.3 mmol/L (50.3 mg/dL)	1.2 mmol/L (46.4 mg/dL)		Acceptable: >1.16 mmol/L (>45 mg/dL) Borderline: 1.03-1.16 mmol/L (40-45 mg/dL) Low: <1.03 mmol/L (<40 mg/dL)

Weight SD and BMI SD were calculated based on the Centers for Disease Control and Prevention growth charts.

Expert Panel on Integrated Guidelines for Cardiovascular Health and Risk Reduction in Children and Adolescents. Expert panel on integrated guidelines for cardiovascular health and risk reduction in children and adolescents: summary report. *Pediatrics.* 2011;128(Suppl 5): S213-S256. doi:10.1542/peds.2009-2107C.

Abbreviations: ALT, alanine transaminase; AST, aspartate transaminase; BMI, body mass index; HbA_1c_, glycated hemoglobin A_1c_; HDL-C, high-density lipoprotein cholesterol; LDL-C, low-density lipoprotein cholesterol; TAG, triacylglycerol; TC, total cholesterol.

Ultrasound scan of the kidneys showed a normal right kidney with normal interval renal growth with mild left hydronephrosis. Magnetic resonance imaging of the brain with magnetic resonance spectroscopy showed nonspecific corpus callosum morphological changes; the intracranial appearances were otherwise unremarkable.

### Case 2: Alström Syndrome

A novel homozygous frameshift pathogenic variant, c.2296_2299delTCAC (p.Ser766Lysfs*13), was identified in the *ALMS1* gene. This variant is predicted to result in nonsense-mediated messenger RNA decay, leading to loss of functional protein.

The patient's kidney function tests and serum electrolytes were within normal limits. Liver enzymes were mildly elevated with alanine transaminase (ALT) 50 IU/L and aspartate transaminase (AST) 38 IU/L.

Glycated hemoglobin A_1c_ (HbA_1c_) was 5.6% (38 mmol/mol) within the normal range. The liver ultrasound showed that the liver was at the upper limit of normal in size, measuring 12.5 cm in length, with normal echogenicity (Table 2).

**Table 2.** Body measurements and laboratory results before treatment and after 7 months of semaglutide therapy in a patient with Alström syndrome and obesity

	Baseline	After 7 mo of treatment	Normal reference range
Weight	52.00 kg, SD +2.18	45.00 kg, SD +0.95	
BMI	26.46, SD +2.09	20.27, SD +1.02	5th-85th percentile (∼14.0-19.0)
HbA_1c_,%	5.6% (38 mmol/mol)	5.3% (34 mmol/mol)	Normal: <5.7% (<39 mmol/mol) Prediabetes: 5.7-6.4% (39-47 mmol/mol) Diabetes: ≥6.5% (≥48 mmol/mol)
ALT	50 IU/L	24 IU/L	<26 IU/L
AST	38 IU/L	24 IU/L	<40 IU/L
TC	5.2 mmol/L (201 mg/dL)	4.7 mmol/L (182 mg/dL)	Acceptable: <4.4 mmol/L (<170 mg/dL) Borderline: 4.4-5.1 mmol/L (170-199 mg/dL) High: ≥5.2 mmol/L (≥200 mg/dL)
TAG	2 mmol/L (177.1 mg/dL)	1.2 mmol/L (106.3 mg/dL)	Acceptable: <1.02 mmol/L (<90 mg/dL) Borderline: 1.02-1.46 mmol/L (90-129 mg/dL) High: ≥1.47 mmol/L (≥130 mg/dL)
LDL-C	3.8 mmol/L (147 mg/dL)	3.4 mmol/L (131 mg/dL)	Acceptable: <2.85 mmol/L (<110 mg/dL) Borderline: 2.85-3.34 mmol/L (110-129 mg/dL) High: ≥3.36 mmol/L (≥130 mg/dL)
HDL-C	1 mmol/L (38.7 mg/dL)	1.3 mmol/L (50.3 mg/dL)	Acceptable: >1.16 mmol/L (>45 mg/dL) Borderline: 1.03-1.16 mmol/L (40-45 mg/dL) Low: <1.03 mmol/L (<40 mg/dL)

Weight SD and BMI SD were calculated based on the Centers for Disease Control and Prevention growth charts.

Expert Panel on Integrated Guidelines for Cardiovascular Health and Risk Reduction in Children and Adolescents. Expert panel on integrated guidelines for cardiovascular health and risk reduction in children and adolescents: summary report. *Pediatrics.* 2011;128(Suppl 5):S213-S256. doi:10.1542/peds.2009-2107C.

Abbreviations: ALT, alanine transaminase; AST, aspartate transaminase; BMI, body mass index; HbA_1c_, glycated hemoglobin A_1c_; HDL-C, high-density lipoprotein cholesterol; LDL-C, low-density lipoprotein cholesterol; TAG, triacylglycerol; TC, total cholesterol.

## Treatment

### Case 1: Bardet-Biedl Syndrome

**Figure 1.**
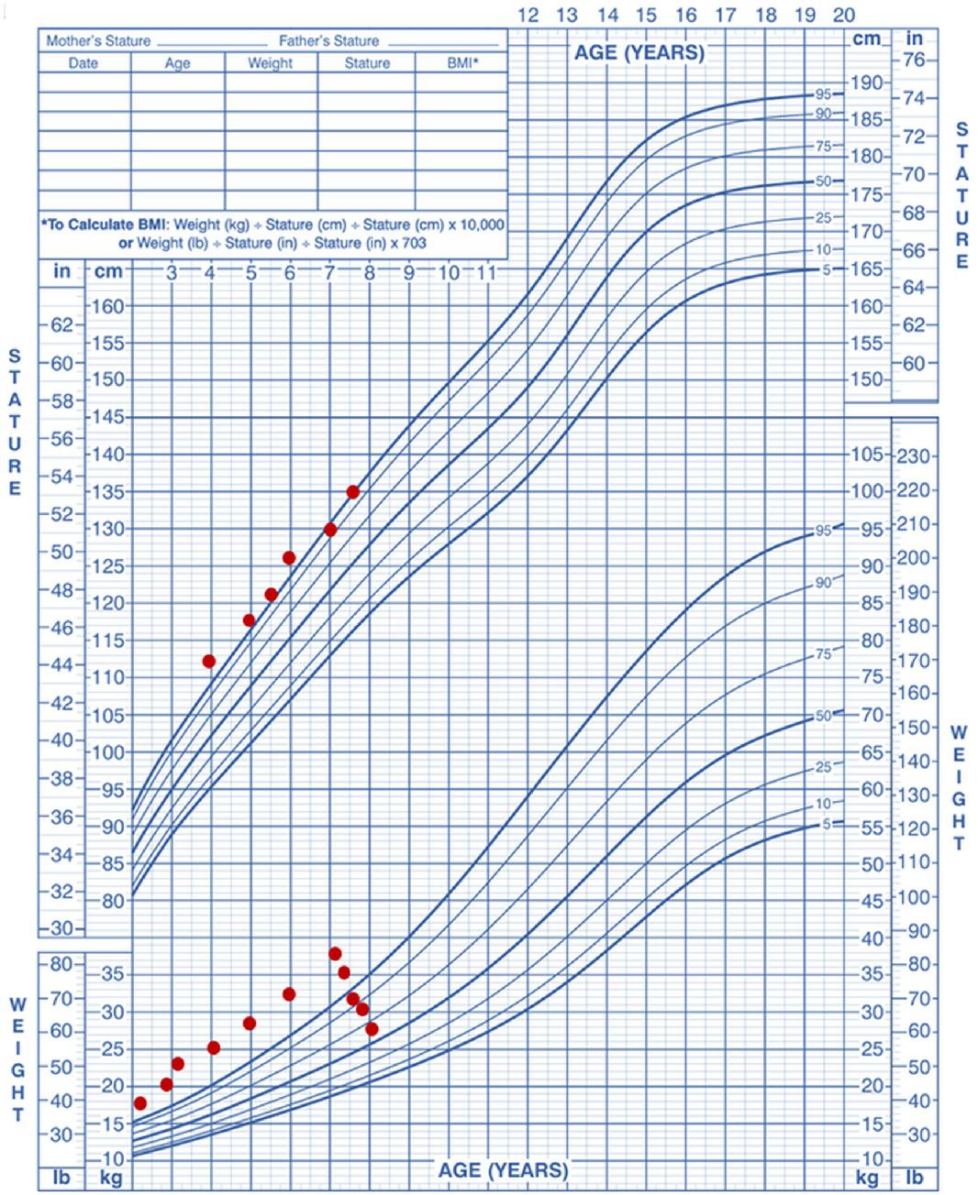
Weight trajectory before and after semaglutide treatment in a patient with Bardet-Biedl syndrome (BBS) and obesity.

The patient had severe hyperphagia, leading to obesity. At age 7 years, he weighed 36.15 kg, with a body mass index (BMI) of 22.1 (SD +2.32; Fig. 1). Given the severity of his hyperphagia, a therapeutic trial was started with once-weekly subcutaneous semaglutide with a dose-escalation schedule (0.5 to 1 mg).

### Case 2: Alström Syndrome

The patient's obesity was managed with dietary modification and exercise, but due to inadequate response, once-weekly subcutaneous semaglutide with a dose-escalation schedule (0.5 to 1 mg) was implemented.

## Outcome and Follow-up

### Case 1: Bardet-Biedl Syndrome

Following this intervention, the patient showed significant weight loss and a reduction in appetite, with no reported adverse effects. On follow-up, 3 months later, his weight had decreased to 31.10 kg, with a BMI of 18.81 (SD +1.47; see Fig. 1). ALT dropped from 31 IU/L to 20 IU/L (normal reference range: <25 IU/L), AST dropped from 31 IU/L to 29 IU/L (normal reference range: <40 IU/L), HBA_1c_ dropped from 5.4% (36 mmol/mol) to 5.0% (31 mmol/mol) (normal reference range: <5.7%), total cholesterol (TC) dropped from 5.0 mmol/L (193 mg/dL) to 4.4 mmol/L (170 mg/dL) (normal reference range: <4.4 mmol/L; <170 mg/dL), and low-density lipoprotein cholesterol (LDL-C) dropped from 3.6 mmol/L (139 mg/dL) to 2.8 mmol/L (108 mg/dL) (normal reference range: <2.85 mmol/L; <110 mg/dL; Table 1).

### Case 2: Alström Syndrome

Since initiating semaglutide, the patient's appetite has markedly decreased. Before treatment, he consumed 3 large meals per day along with frequent high-calorie snacks, including sweets, fried foods, and sugary beverages. He often requested food multiple times between meals and displayed significant food-seeking behaviors. Following the start of semaglutide, he now consumes only one main meal per day, with minimal snacking, and no longer exhibits persistent hunger or food preoccupation. Treatment was well tolerated, without hypoglycemia or gastrointestinal adverse effects.

**Figure 2.**
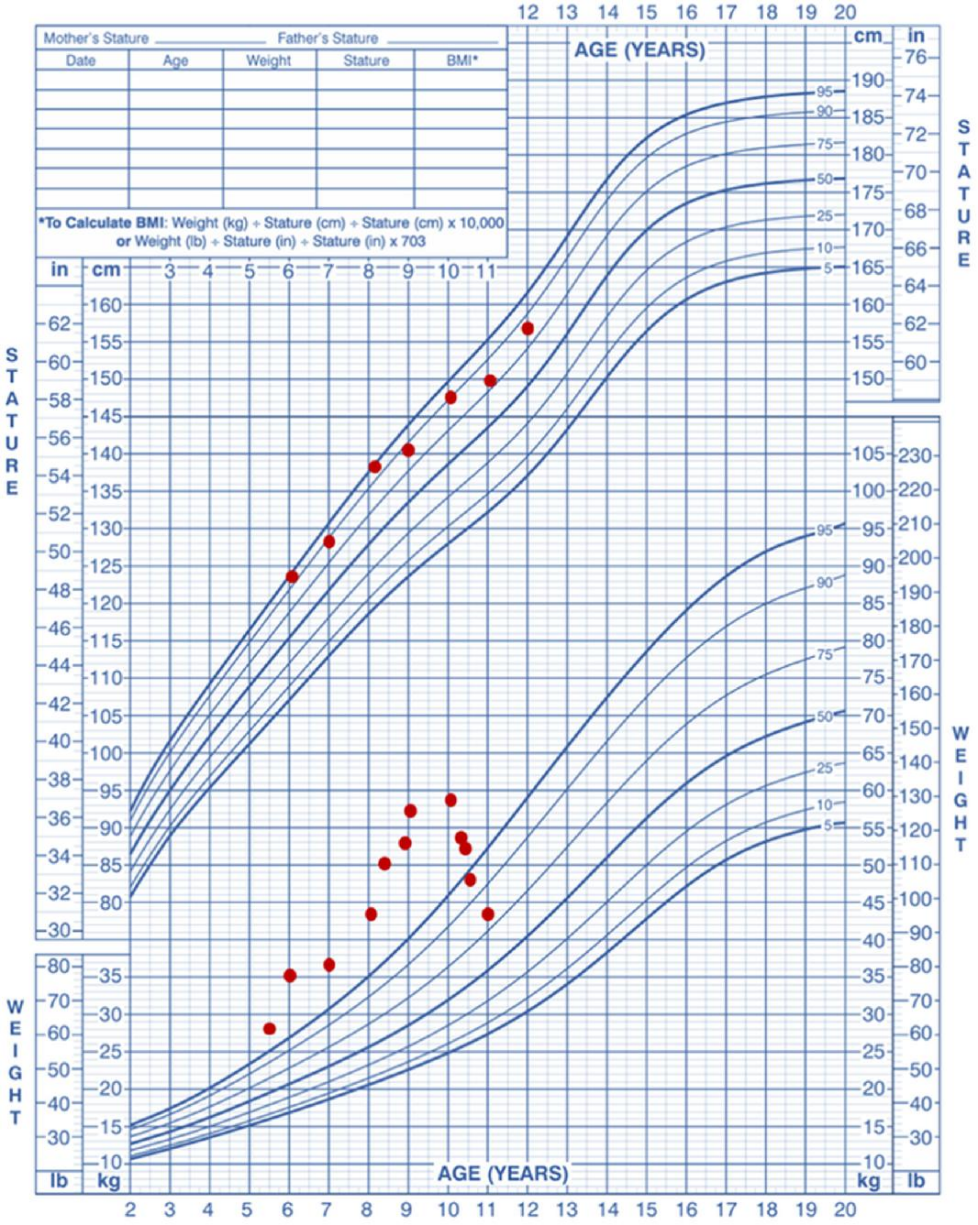
Weight trajectory before and after semaglutide treatment in a patient with Alström syndrome and obesity.

Regular follow-up assessments revealed significant weight loss, from 52.00 kg (SD +2.18, BMI 26.46, SD +2.09) to 45.00 kg (SD 0.95, BMI 20.27, SD 1.02; Fig. 2) over approximately 7 months. HbA_1c_ dropped from 5.6% (38 mmol/mol) to 5.3% (34 mmol/mol) (normal reference range: <5.7%), and ALT dropped from 50 IU/L to 24 IU/L (normal reference range: <25 IU/L). LDL-C dropped from 3.8 mmol/L (147 mg/dL) to 3.4 mmol/L (131 mg/dL) (normal reference range: <2.85 mmol/L; <110 mg/dL; Table 2).

## Discussion

Ciliopathies, such as BBS and AS, contribute to obesity through impaired primary cilia function, which disrupts central energy homeostasis. Primary cilia are essential for hypothalamic neuronal signaling, particularly in the melanocortin pathway involving *MC4R*, where defective ciliary trafficking leads to impaired leptin and satiety signaling, resulting in increased food intake and reduced energy expenditure [1, 9]. In BBS, mutations in *BBS* genes disrupt the BBSome complex, which is critical for trafficking receptors, including leptin and *MC4R*, to neuronal cilia [1]. In AS, *ALMS1* mutations compromise ciliary structure and intracellular trafficking, similarly disturbing receptor signaling and insulin sensitivity [2]. Collectively, these defects predispose affected individuals to early-onset, severe obesity [1, 2, 9].

Setmelanotide and semaglutide represent distinct but complementary approaches to treating obesity in BBS and AS. Setmelanotide specifically targets MC4R pathways and restores leptin signaling [7]. It has demonstrated efficacy in reducing hyperphagia and body weight, with minimal off-target effects, as shown in pivotal clinical trials [8, 9]. However, its high cost remains a barrier to widespread use in both syndromes.

Recent evidence supports the therapeutic potential of glucagon-like peptide-1 receptor agonists (GLP-1RAs) in AS. In a real-world UK cohort, semaglutide or exenatide administered for at least 6 months in adults with genetically confirmed AS led to a mean weight reduction of approximately 6%, a 1.1% drop in HbA_1c_, and improvements in lipid profile and hepatic transaminases—comparable to responses seen in polygenic obesity [10]. Ferch et al [11] further reported two young adults with AS who showed enhanced metabolic response to tirzepatide, a dual glucose-dependent insulinotropic polypeptide/GLP-1RA, after a limited response to semaglutide. The patients achieved weight loss of 7.2% and 26.9%, reduced hepatic steatosis, and improved glycemic control, including an 83% reduction in insulin requirement in one case.

In contrast, data on GLP-1RA therapy in BBS are limited. Ganawa et al [12] reported the case of a 28-year-old woman with BBS who experienced a 33% weight reduction over 30 months on semaglutide. However, long-term metabolic effects and durability of response are yet to be studied in larger cohorts. To date, no randomized controlled trials of GLP-1RAs have been conducted in BBS.

Emerging preclinical data provide further rationale for GLP-1RA use in BBS. In a tamoxifen-inducible Bbs5^−/−^ mouse model [12], semaglutide reversed obesity, improved glycemic control and energy expenditure, and normalized hypothalamic gliosis. The treatment also enhanced pro-opiomelanocortin (POMC) expression, indicating restoration of central satiety signaling. Similarly, in Bbs1^M390R/M390R^ mice, Tomlinson [13] showed that semaglutide reduced visceral adiposity and improved insulin sensitivity, with upregulation of hypothalamic GLP-1R expression, suggesting both peripheral and central therapeutic effects.

Semaglutide's mechanism involves the modulation of several metabolic pathways. It enhances satiety through hypothalamic signaling, activating anorexigenic POMC/CART neurons and inhibiting orexigenic neuropeptide Y/agouti-related peptide neurons via the cyclic adenosine monophosphate (cAMP)–protein kinase A (PKA) and PI3K–protein kinase B pathways [9]. In pancreatic β cells, semaglutide increases intracellular cAMP, activating PKA and Epac2, promoting insulin secretion via PI3 K/mechanistic target of rapamycin pathways [14]. Additionally, it exerts anti-inflammatory and cardioprotective effects through adenosine monophosphate–activated protein kinase C and SIRT1 activation, which inhibit nuclear factor κB signaling and reduce oxidative stress [15].

While setmelanotide provides a targeted intervention for MC4R pathway defects, its mutation-specific nature and cost limit its real-world applicability. In contrast, semaglutide is more broadly effective, affordable, and accessible, making it a practical choice for syndromic obesity with multisystem involvement.

In this paper, we describe the first clinical experience using semaglutide in a child with BBS and AS, demonstrating favorable outcomes in weight, glycemic control, and hepatic steatosis. These early findings support consideration of GLP-1RA therapy in BBS and reinforce its established utility in AS. Further studies and clinical trials are warranted to evaluate its efficacy, safety, and long-term metabolic effect in both conditions.

## Learning Points

- Semaglutide is affordable, broadly effective, and accessible, with multisystem benefits in pediatric obese children with BBS and AS.
- Treatment choice should be individualized; combination strategies may offer synergistic benefits.

## Contributors

H.D. and K.H. managed the patients and prepared the manuscript. I.M. contributed to genetic assessment and interpretation. All authors reviewed and approved the final version.

## Data Availability

Original data generated and analyzed during this study are included in this published article.

## Abbreviations

ALT: alanine transaminase
AS: Alström syndrome
AST: aspartate transaminase
BBS: Bardet-Biedl syndrome
BMI: body mass index
cAMP: cyclic adenosine monophosphate
GLP-1: glucagon-like peptide-1
GLP-1RAs: glucagon-like peptide-1 receptor agonists
HbA_1c_: glycated hemoglobin A_1c_
HDL-C: high-density lipoprotein cholesterol
LDL-C: low-density lipoprotein cholesterol
MC4R: melanocortin-4 receptor
PKA: protein kinase A
POMC: pro-opiomelanocortin
T2DM: type 2 diabetes mellitus
TAG: triacylglycerol
TC: total cholesterol

## References

[1] Lee CH, Kang GM, Kim MS. Mechanisms of weight control by primary cilia. Mol Cells. 2022;45(4):169‐176. 35387896 10.14348/molcells.2022.2046 PMC9001153

[2] Dollfus H, Lilien MR, Maffei P, et al Bardet-Biedl syndrome improved diagnosis criteria and management: inter European reference networks consensus statement and recommendations. Eur J Hum Genet. 2024;32(11):1347‐1360. 39085583 10.1038/s41431-024-01634-7 PMC11576898

[3] Tahani N, Maffei P, Dollfus H, et al Consensus clinical management guidelines for Alström syndrome. Orphanet J Rare Dis. 2020;15(1):253. 32958032 10.1186/s13023-020-01468-8 PMC7504843

[4] Niederlova V, Modrak M, Tsyklauri O, Huranova M, Stepanek O. Meta-analysis of genotype-phenotype associations in Bardet-Biedl syndrome uncovers differences among causative genes. Hum Mutat. 2019;40(11):2068‐2087. 31283077 10.1002/humu.23862

[5] Forsythe E, Sparks K, Best S, et al Risk factors for severe renal disease in Bardet-Biedl syndrome. J Am Soc Nephrol. 2017;28(3):963‐970. 27659767 10.1681/ASN.2015091029 PMC5328148

[6] Markham A . Setmelanotide: first approval. Drugs. 2021;81(3):397‐403. 33638809 10.1007/s40265-021-01470-9

[7] Haws RM, Brady S, Davis E, et al Effect of setmelanotide, a melanocortin-4 receptor agonist, on obesity in Bardet-Biedl syndrome and Alström syndrome: results from two phase 3 trials. Nat Med. 2020;26(5):709‐718. 10.1111/dom.14133 PMC7689750 32627316

[8] Haqq AM, Kaplan J, Baker R, et al Long-term efficacy and safety of setmelanotide in individuals with Bardet-Biedl or Alström syndrome: results from the pivotal phase 3 trials. Lancet Diabetes Endocrinol. 2022;10(12):859‐868. 36356613 10.1016/S2213-8587(22)00277-7 PMC9847480

[9] Müller TD, Finan B, Bloom SR, et al Glucagon-like peptide 1 (GLP-1). Nat Rev Drug Discov. 2019;18(12):799‐823.

[10] Ali S, Baig S, Wanninayake S, et al Glucagon-like peptide-1 analogues in monogenic syndromic obesity: real-world data from a large cohort of alström syndrome patients. Diabetes Obes Metab. 2024;26(3):989‐996. 38151964 10.1111/dom.15398

[11] Ferch M, Peitsch I, Kautzky-Willer A, et al Effectiveness of the dual GIP/GLP-1 agonist tirzepatide in two cases of Alström syndrome, a rare obesity syndrome. J Clin Endocrinol Metab. Published online April 30 2025. Doi:10.1210/clinem/dgaf258 PMC12623033 40302276

[12] Ganawa S, Sadoul JL, Ezzat S, et al GLP-1RA therapy in Bardet-Biedl syndrome: a case report. Endocr Abstr. 2022;82:WG1.

[13] Tomlinson JW . Commentary: targeting GLP-1 receptors in Bardet-Biedl syndrome—potential and limitations. J Clin Invest. 2025;135(12):e191822. 40519161 10.1172/JCI191822 PMC12165807

[14] Shigeto M, Ramracheya R, Tarasov AI, et al GLP-1 stimulates insulin secretion via Ca²⁺-independent cyclic AMP signaling pathways in pancreatic β-cells. Diabetes. 2015;64(4):1273‐1283.25352639

[15] Lee YS, Jun HS. Anti-inflammatory effects of GLP-1-based therapies beyond glucose control. Mediators Inflamm. 2016;2016:3094642. 27110066 10.1155/2016/3094642 PMC4823510

